# Correction to “Sarcopenia Definitions and Diagnostic Challenges Across Diverse Clinical Populations: A Scoping Review”

**DOI:** 10.1002/hsr2.73454

**Published:** 2026-11-09

1

Sarcopenia Definitions and Diagnostic Challenges Across Diverse Clinical Populations: A Scoping Review. Health Science Reports. DOI: 10.1002/hsr2.72782.

The affiliation of the author Minoo Heidari Almasi was incorrect and has been corrected to “Clinical Research Development Center, Imam Hossein Educational Hospital, Shahid Beheshti University of Medical Sciences, Tehran, Iran.”

The Online Version of This Article Has Been Corrected Accordingly

We apologize for this error.

